# Sulfur source-mediated transcriptional regulation of the *rhlABC* genes involved in biosurfactants production by *Pseudomonas* sp. strain AK6U

**DOI:** 10.3389/fmicb.2014.00423

**Published:** 2014-08-14

**Authors:** Wael Ismail, Ashraf M. El Nayal, Ahmed R. Ramadan, Nasser Abotalib

**Affiliations:** Biotechnology Program, College of Graduate Studies, Arabian Gulf UniversityManama, Bahrain

**Keywords:** gene expression, surface tension, RT-qPCR, rhamnolipids, dibenzothiophene, biodesulfurization

## Abstract

Despite the nutritional significance of sulfur, its influence on biosurfactants production has not been sufficiently studied. We investigated the expression of key biosurfactants production genes, *rhlABC*, in cultures of *Pseudomonas* sp. AK6U grown with inorganic or organic sulfur sources. AK6U grew with either inorganic sulfate (MgSO_4_), dibenzothiophene (DBT), or DBT-sulfone as a sole sulfur source in the presence of glucose as a carbon source. The AK6U cultures produced variable amounts of biosurfactants depending on the utilized sulfur source. Biosurfactants production profile of the DBT cultures was significantly different from that of the DBT-sulfone and inorganic sulfate cultures. The last two cultures were very similar in terms of biosurfactants productivity. Biosurfactants yield in the DBT cultures (1.3 g/L) was higher than that produced by the DBT-sulfone (0.5 g/L) and the inorganic sulfate (0.44 g/L) cultures. Moreover, the surface tension reduction in the DBT cultures (33 mN/m) was much stronger than that measured in the DBT-sulfone (58 mN/m) or inorganic sulfate (54 mN/m) cultures. RT-qPCR revealed variations in the expression levels of the *rhlABC* genes depending on the sulfur source. The DBT cultures had higher expression levels for the three genes as compared to the DBT-sulfone and inorganic sulfate cultures. There was no significant difference in the expression profiles between the DBT-sulfone and the MgSO_4_ cultures. The increased expression of *rhlC* in the DBT cultures is indicative for production of higher amounts of dirhamnolipids compared to the DBT-sulfone and inorganic sulfate cultures. The gene expression results were in good agreement with the biosurfactants production yields and surface tension measurements. The sulfur source mediates a fine-tuned mechanism of transcriptional regulation of biosurfactants production genes. Our findings can have an impact on industrial production of biosurfactants and other biotechnological processes like biodesulfurization.

## INTRODUCTION

Biosurfactants are surface active natural compounds produced by many microorganisms. As compared to petrochemicals-derived (synthetic) surfactants, biosurfactants are characterized by superior physicochemcial properties in addition to their environmental compatibility (Desai and Banat, 1997). They are structurally diverse, amphipathic, and can lower surface and interfacial tension. An effective biosurfactant can reduce the surface tension of water from 72 to 35 mN/m (Soberón-Chávez and Maier, 2011). They can also emulsify various hydrocarbons (Sekhon et al., 2012).

Biosurfactants have attracted an increasing interest as efficient and eco-friendly substitutes to synthetic surfactants in many environmental, industrial, agricultural, and biomedical applications. These include, bioremediation (biodegradation), soil washing, biocontrol and spray application of fertilizers, enhanced oil recovery, de-emulsification, cosmetics, pharmaceuticals, antimicrobial agents, foods, beverages, etc. (Urum and Pekdemir, 2004; Mulligan, 2005; Rodrigues et al., 2006; Perfumo et al., 2010; Rodrigues and Teixeira, 2010).

Although there has been an increasing number of reports describing the production and characterization of efficient biosurfactants, to date biosurfactants are still not able to economically compete with synthetic surfactants. This is mainly due to high production costs (Cameotra and Makkar, 1998; Deleu and Paquot, 2004). One way to reduce the prohibitive high costs of biosurfactants is to enhance the strain productivity by, for instance, optimizing the growth conditions. The carbon source in the growth medium is of particular interest. Inexpensive carbon sources have been used for biosurfactants production to minimize the overall production costs.

Many studies reported improved biosurfactants yield with the application of industrial carbon-rich wastes (Makkar et al., 2011; Merchant and Banat, 2012). Various cheap substrates have the potential of enhancing biosurfactants production. These include vegetable oils, and oil wastes, animal fat, molasses, lactic whey, starchy substrates, etc. (Haba et al., 2000; Abalos et al., 2001; Dubey et al., 2005; Sekhon et al., 2012).

In contrast to the carbon source, the effect of the sulfur source on biosurfactants production has not received the proper attention, although it is an essential component of the growth medium. In an earlier study, Pruthi and Cameotra (2003) reported that 80 ppm of inorganic sulfur was the optimal concentration for biosurfactants production by *Pseudomonas putida*. However, the authors did not test different types of sulfur sources and did not discuss the observed effect of sulfur concentration on biosurfactants production. Recently we have isolated a *Pseudomonas* sp. strain AK6U which can simultaneously produce rhamnolipid biosurfactants and utilize organosulfur compounds as sole sulfur sources. Interestingly, we have noticed that biosurfactants production was significantly enhanced in cultures containing organosulfur substrates as compared to cultures containing inorganic sulfate as a sole sulfur source (unpublished). Consequently, an interesting question arose. Is the increase in biosurfactants production due to promotion in the expression of the relevant genes or enhanced activity of the involved biosynthetic enzymes?

Here we conducted further investigations to unravel the reason behind the observed increase in biosurfactants production. We wanted to find out if it is due to enhanced expression of the *rhlABC* genes which encode key enzymes of rhamnolipid biosurfactants biosynthesis. RhlA catalyzes the synthesis of hydroxyalkanoic acid dimers which represent the hydrophobic moiety of rhamnolipid biosurfactants produced by many *Pseudomonas* spp. RhlB and RhlC are rhamnosyltransferases which catalyze the transfer of dTDP-L-rhamnose to either hydroxyalkanoic acid moiety (to produce monorhamnolipid) or to an existing monorhamnolipid molecule to produce dirhamnolipid, respectively (Deziel et al., 2003). We adopted real time quantitative polymerase chain reaction (RT-qPCR) with gene specific primers to investigate the expression of the *rhlABC* genes in the presence of different sulfur sources.

## MATERIALS AND METHODS

### CULTURE MEDIA AND THE BACTERIAL STRAIN

The AK6U strain was isolated from soil polluted with diesel, benzene, and used lubricating oil. The strain was recovered from a mixed culture enriched in chemically defined medium (CDM) containing glucose as a carbon source and DBT as a sulfur source (unpublished). Lauria-Bertani (LB) agar and broth media were prepared according to the instructions of the supplier. Sulfur-free CDM was prepared from stock solutions according to Gilbert et al. (1998). The CDM was supplemented with vitamins solution and trace elements (Pfenning, 1978; Van Hamme et al., 2000). The carbon source was glucose (10 mM) and the sulfur source was either MgSO_4_ ⋅ 7H_2_O (1 mM) or an organosulfur compound (0.1 mM). The tested organosulfur substrates were dibenzothiophene (DBT) and dibenzothiophene sulfone (DBT-sulfone). DBT is one of the most common organosulfur compounds found in petroleum and diesel. It is also the most frequently adopted model compound for the biodesulfurization studies (Monticello and Finnerty, 1985; Monot and Warzywoda, 2008). DBT-sulfone is an intermediate of the biodesulfurization 4S pathway (Monot and Warzywoda, 2008). Stock solutions of the organosulfur compounds were prepared in ethanol (100 mM for DBT) or acetone (50 mM for DBT-sulfone). MgCl_2_ (1 mM) was added instead of MgSO_4_ when organosulfur compounds were added as a sole sulfur source.

### GROWTH OF AK6U ON DIFFERENT SULFUR SOURCES AND BIOSURFACTANTS PRODUCTION

Precultures were grown in sulfur-free CDM (100 mL in 250 mL Erlenmeyer flasks) containing glucose as a carbon source and either MgSO_4_ or an organosulfur compound as a sole sulfur source. At the mid-log phase, culture samples were drawn and inoculated into 400 mL of the same medium in 1 L Erlenmeyer flasks (in triplicates). The inoculum size was 1–2% v/v (6–8 mg dry cell weight/L). Growth was monitored by measuring culture turbidity (optical density at 600 nm, OD_600_) after time intervals until the cultures reached the stationary phase. Uninoculated flasks containing the same medium were included as controls. The biomass yield was measured as dry cell weight by drying cell pellets at 105°C for 15 h. All liquid cultures were incubated in an orbital shaker (180 rpm) at 30°C. Culture foaming was monitored as a preliminary indication of biosurfactants production. Cells were harvested at the late exponential growth phase by centrifugation in pre-cooled centrifuge at 10,000 rpm for 10 min (Beckman centrifuge J2–21, USA). Cell pellets were washed once with 0.1 M ice–cold phosphate buffer (pH 7) and the washed cell pellets were stored at -20°C. All the cell harvesting procedures were performed on ice.

### HIGH PERFORMANCE LIQUID CHROMATOGRAPHY (HPLC)

Cultures grown on different sulfur sources were analyzed by high performance liquid chromatography (HPLC) to monitor the utilization of the organosulfur compounds. Culture samples (500 μL) were centrifuged at 14,000 rpm for 5 min and the cell-free supernatants were extracted once with one volume of ethylacetate. The organic phase (300 μL of ethylacetate) was removed and transferred to clean (1.5 mL) eppendorf tubes. Ethylacetate was evaporated in a vacuum concentrator (speed-vac) at room temperature. The residue was dissolved in 50 μL ethanol and analyzed using a Thermo-Dionex UHPLC 3000 (Thermo, USA), and an Acclaim^TM^ 120 C18 column (5 μm, 120 A, Thermo, USA). The mobile phase was 60% acetonitrile-water pumped at a flow rate of 1 mL/min. Detection was performed with a photodiode array at 233 and 248 nm.

### MEASUREMENT OF SURFACE TENSION

Culture samples (20 mL) were retrieved after time intervals and the cells were removed by centrifugation for 5 min at 10,000 rpm (4°C). The cell-free supernatants were then filtered under vacuum through 0.22 μm membrane filters to remove residual cells. The surface tension of cell-free culture supernatants was measured with a Kruss K100MK3 tensiometer (Kruss, Germany) equipped with a platinum plate at room temperature via the Wilhelmy plate method (Walter et al., 2010). The instrument was calibrated by adjusting the measurement so that the surface tension of water is 72 mN/m at room temperature.

### RECOVERY OF THE CRUDE BIOSURFACTANTS

Cell-free culture supernatants (100 mL) were collected by centrifugation (14,000 rpm, 10 min) at the late-log growth phase and filtered through 0.22 μm membrane filters (Millipore, USA) to remove residual cells. After acidification to pH 2 with 25% HCl, the supernatants were kept at 4°C overnight. The biosurfactants were extracted twice with one volume of chloroform-methanol (2:1) in a separating funnel. The organic phases were pooled and evaporated under vacuum (Buchi rotary evaporator V850, Switzerland) at 40°C. The residue was weighed and the crude biosurfactants yield was estimated as g/L.

### ISOLATION OF RNA AND cDNA SYNTHESIS

Total RNA was isolated from 0.003 g ± 0.0001 of cell pellets harvested from different cultures using RNeasy Mini kit (Qiagen, Germany). The isolated RNA was treated twice with DNaseI. One treatment was done on-column and the second treatment was performed on the eluted RNA. Agarose gel electrophoresis (0.7%) was used to check the quality of RNA. The concentration and purity of RNA were estimated using Biophotometer plus (Eppendorf, Germany). To check DNA contamination, we performed PCR with gene-specific primers (**Table 1**) using the isolated RNA as a template. The PCR conditions were: 5 min at 95°C followed by 40 cycles of 30 s at 95°C, 30 s at 58°C, 30 s at 72°C, and final extension step 5 min at 72°C. cDNA was synthesized from a normalized RNA quantity (800 ng) with High Capacity cDNA Reverse Transcription kit (ABI, USA) according to manufacturer’s instructions. cDNA was used as a template in RT-qPCR assays as explained in the following.

**Table 1:** Primers used in this study.

Primer name	Primer sequence (5′–3′)	Target gene	Product size (bp)	Reference
16S-F 16S-R	CACCGGCAGTCTCCTTAGAG AAGCAACGCGAAGAACCTTA	16S rRNA	203	This study
rhlA-F rhlA-R	TGGACTCCAGGTCGAGGAAA GAAAGCCAGCAACCATCAGC	*rhlA*	263	This study
Kpd1 Kpd2	GCCCACGACCAGTTCGAC CATCCCCCTCCCTATGAC	*rhlB*	226	Bodour et al. (2003)
rhlC-F2 rhlC-R2	GTCGAGTCCCTGGTTGAAGG CGTGCTGGTGGTACTGTTCA	*rhlC*	211	This study

### RT-qPCR

The primers used in this study are shown in **Table 1**. Primers specific for the *rhlAC* genes were derived from the corresponding sequences harbored in the genome of the *Pseudomonas aeruginosa* PAO1 strain (Genbank accession number NC_002516). Primers specific for the 16S rRNA gene were designed based on the partial sequence of the 16S rRNA gene of the AK6U strain (Genbank accession number AB922602). Delta–delta C_T_ relative quantification of gene expression was used to investigate the change in gene expression in cultures grown with different sulfur sources. 16S rRNA gene was included as an endogenous control and the results were expressed as fold change in expression. For each condition, three cDNA preparations (obtained from three independent cultures) were tested in RT-qPCR. For each cDNA replicate, two RT-qPCR assays were performed using Rotor-Gene Q (Qiagen, Germany). Each RT-qPCR assay (20 μL) contained 10 μL Rotor-Gene master mix (SYBR-Green PCR kit, Qiagen, Germany), 0.5 μM of each primer, 1 μL of cDNA and the rest was completed with nuclease-free water. PCR conditions were five minutes at 95°C followed by 40 cycles of 15 s at 95°C, 20 s at 58°C, and 30 s at 60°C.

### STATISTICAL ANALYSIS

One way analysis of variance (Tukey test with *p* < 0.05) was performed with the JMP statistical software (version 10.0.2, SAS Corporation, Chicago, IL, USA).

## RESULTS

### BIOSURFACTANTS PRODUCTION BY AK6U UTILIZING DIFFERENT SULFUR SOURCES

**FIGURE 1:**
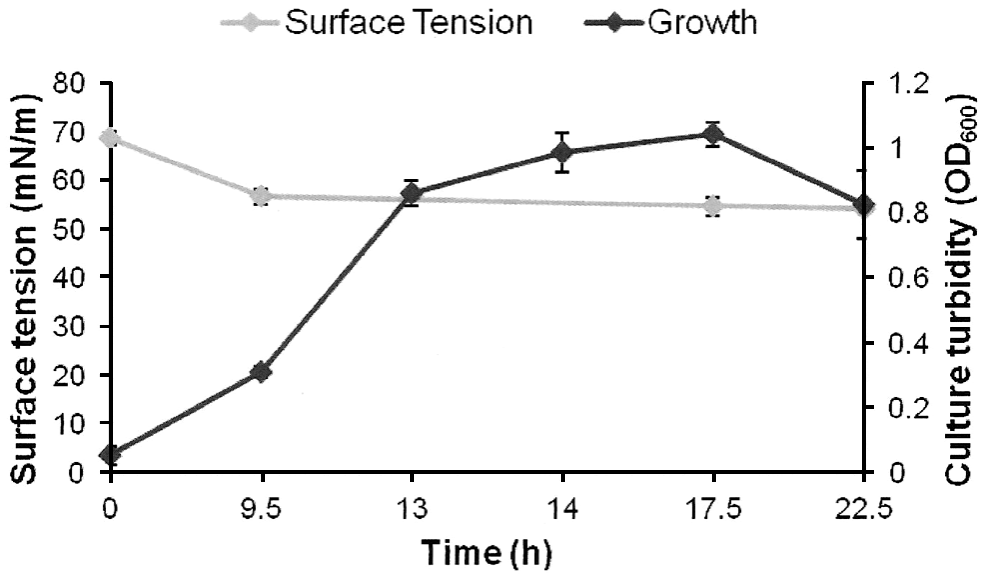
**Growth and biosurfactants production by AK6U in sulfur-free minimal medium containing MgSO_**4**_ as a sole sulfur source and glucose as a carbon source.** Biosurfactants production was monitored by measuring the surface tension of the cell-free culture supernatants. Data are averages of measurements from three cultures ± SE.

**FIGURE 2:**
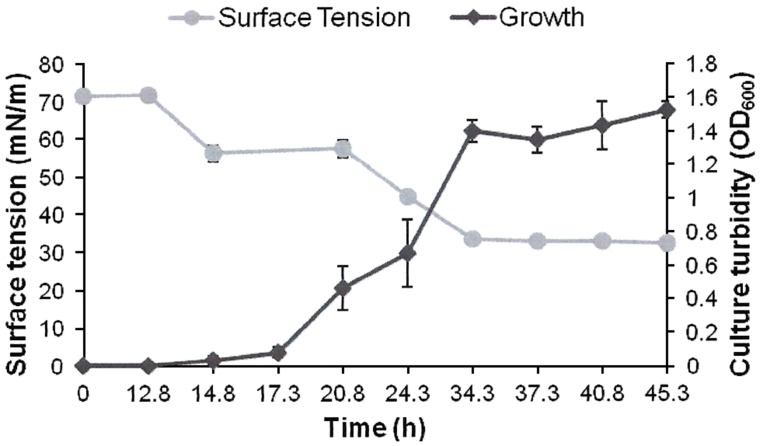
**Growth and biosurfactants production by AK6U in sulfur-free minimal medium containing DBT as a sole sulfur source and glucose as a carbon source.** Biosurfactants production was monitored by measuring the surface tension of the cell-free culture supernatants. Data are averages of measurements from three cultures ± SE.

**FIGURE 3:**
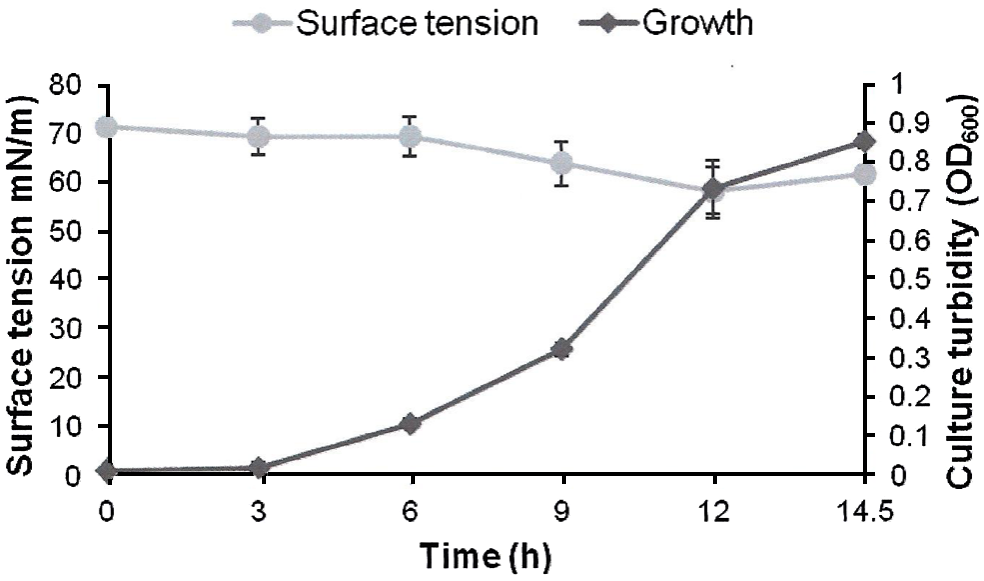
**Growth and biosurfactants production by AK6U in sulfur-free minimal medium containing DBT-sulfone as a sole sulfur source and glucose as a carbon source.** Biosurfactants production was monitored by measuring the surface tension of the cell-free culture supernatants. Data are averages of measurements from three cultures ± SE.

**FIGURE 4:**
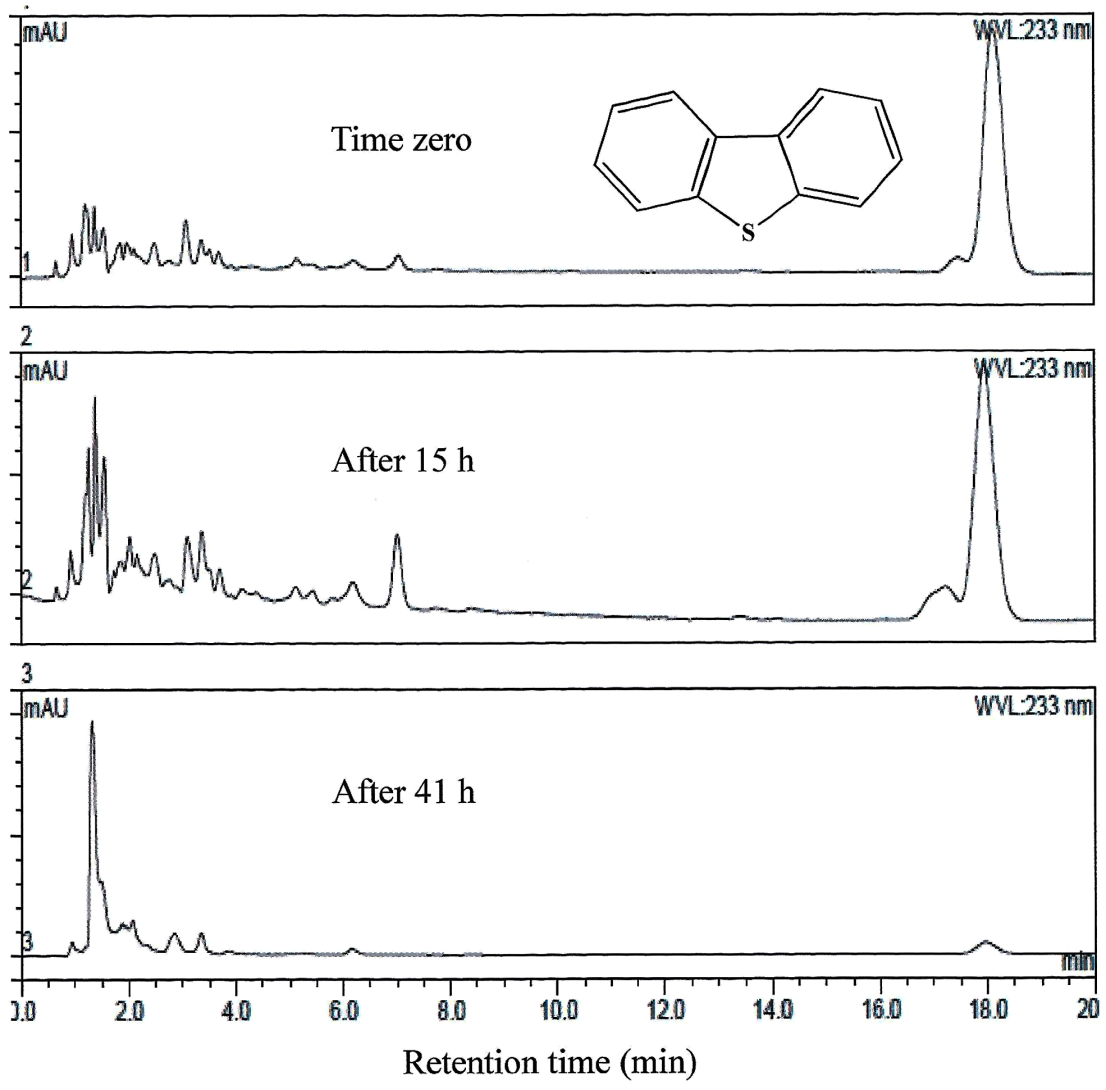
**HPLC analysis showing the utilization of DBT by AK6U grown in sulfur-free minimal medium containing DBT as a sole sulfur source and glucose as a carbon source.** The chemical structure of DBT is shown and refers to the major peak in the chromatogram (retention time 18 min).

**FIGURE 5:**
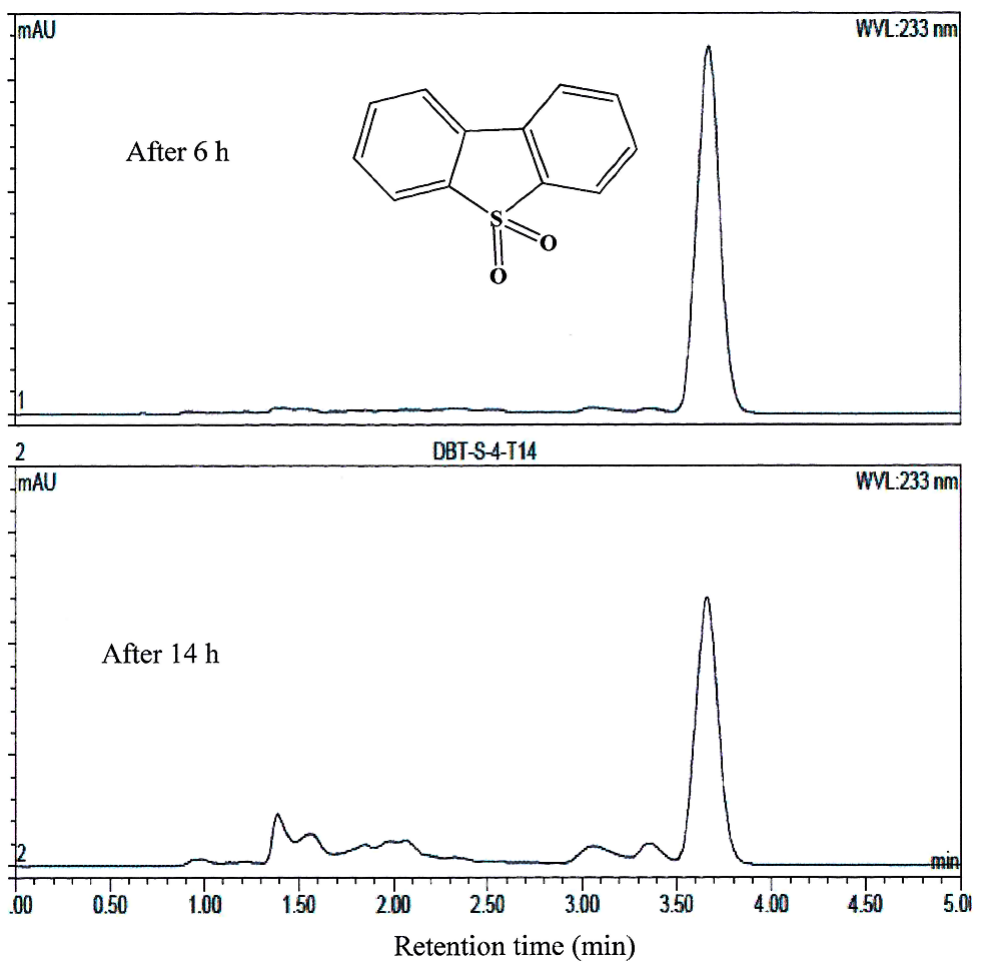
**HPLC analysis showing the utilization of DBT-sulfone by AK6U grown in sulfur-free minimal medium containing DBT-sulfone as a sole sulfur source and glucose as a carbon source.** The chemical structure of DBT-sulfone is shown and refers to the major peak in the chromatogram (retention time 3.7 min).

AK6U grew in minimal medium containing either MgSO_4_, DBT, or DBT-sulfone as a sole sulfur source in the presence of glucose as a carbon source (**Figures 1–3**). HPLC analysis of culture samples after time intervals revealed the utilization of the organosulfur substrates DBT and DBT-sulfone (**Figures 4** and **5**). Biosurfactants production was indicated by culture foaming and changes in the surface tension of the growth medium over time. The DBT cultures exhibited stronger foaming than either the MgSO_4_ or the DBT-sulfone cultures that revealed similar foaming profile.

The surface tension was measured in cell-free culture supernatants. Interestingly, the surface tension decreased with time in all cultures (**Figures 1–3**). However, the extent of surface tension reduction was different depending on the utilized sulfur source (**Table 2**). The surface tension of the DBT culture was significantly different from that of the DBT-sulfone and the MgSO_4_ cultures (*p* < 0.05). There was no significant difference in surface tension between the DBT-sulfone and the MgSO_4_ cultures (*p* > 0.05). The DBT cultures recorded the strongest reduction in surface tension (from 72 to 33 mN/m). In contrast, surface tension reduction in the MgSO_4_ and DBT-sulfone cultures was much weaker. The minimal surface tension in the MgSO_4_ culture was 54 mN/m, whereas that of the DBT-sulfone cultures was 58 mN/m. The lowest surface tension in the DBT culture was attained towards the end of the exponential growth (after 25 h of incubation). The MgSO_4_ and DBT-sulfone cultures reached the minimal surface tension value in a shorter time. This was also towards the end of exponential growth in all cultures. No further reduction in surface tension was observed during the stationary growth phase.

**Table 2:** Characteristics of biosurfactants production by AK6U.

Sulfur source	Minimal surface tension^∗^ (mN/m)	Biosurfactants yield (g/L)
MgSO_4_	54.3 ± 0.2 A	0.44
DBT	32.8 ± 0.2 B	1.3
DBT-sulfone	58.2 ± 0.7 A	0.5

*^∗^Data are means of three replicates ± SE. Values labeled with different letters are significantly different (*p* < 0.05).*

A crude form of the produced biosurfactants was recovered from cell-free culture supernatants via acidification and solvent extraction. In addition to the variability in surface tension reduction, the AK6U cultures produced different amounts of biosurfactants depending on the utilized sulfur source (**Table 2**). The DBT cultures produced the highest biosurfactants yield (1.3 g/L). This was ca threefold higher than that recovered from the MgSO_4_ and DBT-sulfone cultures. The latter two cultures produced similar quantities of crude biosurfactants.

### RNA ISOLATION AND cDNA SYNTHESIS

Total RNA was successfully isolated from cell pellets harvested from different AK6U cultures. The integrity of the isolated RNA was verified by gel electrophoresis. The second DNaseI treatment removed genomic DNA contamination as revealed by gel electrophoresis. This was confirmed by using the isolated RNA as a template with gene-specific (*rhlB*) primers in PCR. This PCR failed to give any amplicons which confirms the absence of genomic DNA. cDNA was synthesized from the isolated RNA and was used as a template in PCR also with primers specific for the *rhlB* gene. Specific bands with the expected size were visible on the agarose gels.

### EXPRESSION OF THE *rhlABC* GENES

**FIGURE 6:**
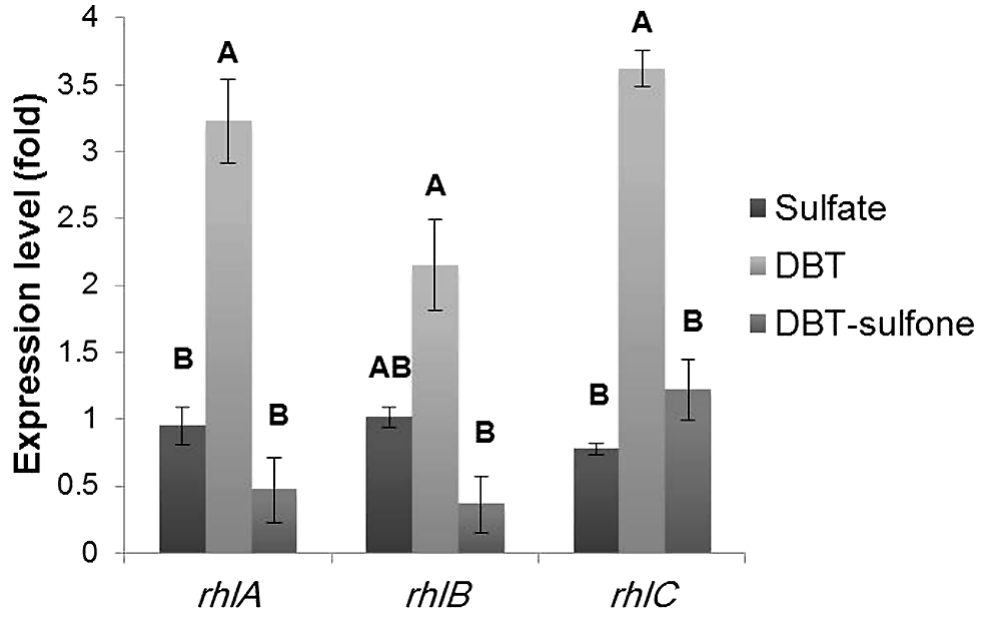
**Results of RT-qPCR showing the relative expression levels of the *rhlABC* genes in AK6U cultures grown with different sulfur sources in the presence of glucose as a carbon source.** The expression level was calculated relative to that of the MgSO_4_ culture as a control group. Data are means of at least two replicates ± SE. Significantly different values (*p* < 0.05) are indicated by different letters above the bars.

We applied RT-qPCR to investigate the influence of the sulfur source on the expression of the *rhlABC* genes in AK6U cultures grown on different sulfur sources. As shown in **Figure 6**, the *rhlABC* genes expression profile was different among the AK6U cultures depending on the utilized sulfur source. In general, the DBT culture revealed the highest expression level for the three genes. The difference in gene expression levels between the DBT culture on one hand and the MgSO_4_ and DBT-sulfone cultures, on the other hand, was statistically significant (*p* < 0.05). However, there was no significant difference in gene expression level between the MgSO_4_ and the DBT-sulfone cultures (*p* > 0.05).

As compared to the MgSO_4_ culture, the DBT culture had 3.2-fold higher expression for *rhlA*, 2.15-fold higher expression for *rhlB*, and 3.6-fold higher expression for *rhlC* (**Figure 6**). Compared to the DBT-sulfone culture, the DBT culture had 5.9-fold higher expression for *rhlA*, 5.3-fold higher expression for *rhlB*, and 3-fold higher expression for *rhlC*. The lowest expression level of the *rhlAB* genes was measured in the DBT-sulfone cultures. Moreover, the expression level of each of the *rhlABC* genes was variable in the same culture. For instance, in the DBT culture, the expression level of *rhlA* and *rhlC* was higher than that of *rhlB*.

Interestingly, the *rhlC* expression level in the DBT-sulfone culture was 1.2-fold higher than that of *rhlA*. There was no significant difference between the expression levels of *rhlA* and *rhlB* in the DBT-sulfone culture (*p* > 0.05). In the MgSO_4_ culture, the expression level of *rhlB* (1.01) was significantly different from that of *rhlC* (0.77).

## DISCUSSION

Both inorganic sulfate and organosulfur substrates were adopted to test their effect on the expression of the *rhlABC* genes. The tested organosulfur compounds, namely DBT and DBT sulfone, are structurally similar and differ in their polarity (hydrophilicity). DBT-sulfone is more polar than DBT due to the presence of the two oxygen atoms (sulfonyl group). Growth of AK6U on either DBT or DBT-sulfone as a sole sulfur source indicates its ability to obtain sulfur from these two substrates in a process known as biodesulfurization (Gallagher et al., 1993; Chen et al., 2008).

The observed culture foaming is a preliminary indication of biosurfactants production (Ismail et al., 2013; Pasternak and Kotwzan, 2013). Results of the surface tension measurements were in agreement with the culture foaming trends. The DBT culture had the lowest surface tension. This provides a direct evidence for biosurfactants production. It further confirms that the DBT cultures produced much more biosurfactants than the DBT-sulfone or the MgSO_4_ cultures. Ibacache-Quiroga et al. (2013) reported biosurfactants production by *Cobetia* sp. growing with DBT, however, as a carbon and energy source. It appears that the DBT-sulfone and the MgSO_4_ cultures have similar biosurfactants productivity. This was inferred from the similar foaming and surface tension profiles. The extraction of more biosurfactants from the DBT culture is in line with suggestion that it produces higher amounts of biosurfactants as compared to the DBT-sulfone and the MgSO_4_ cultures. Altogether, the results clearly point to the influence of the sulfur source on biosurfactants production. Moreover, biosurfactants appear to play a significant role in the biodesulfurization of organosulfur compounds like DBT (Amin et al., 2013; Dinamarca et al., 2014).

The differences in biosurfactants production by AK6U can be attributed to variations in the expression profiles of the *rhlABC* genes. That is the answer to the main question of this study. RT-qPCR revealed different expression patterns among the AK6U cultures depending on the utilized sulfur source. This clearly shows that the sulfur source plays a regulatory role in the expression of the biosurfactants-related genes *rhlABC*. Upregulation of *rhlC* expression in the DBT and DBT-sulfone cultures indicates that they produce more dirhamnolipids than the MgSO_4_ cultures. Interestingly, the gene expression profiles are in very good agreement with and corroborate the results of the surface tension measurements, biosurfactants yield, and culture foaming trends. Moreover, all support the suggested involvement of biosurfactants as a facilitator of DBT biodesulfurization.

Biosurfactants production is known to be controlled by a sophisticated and highly organized regulatory network at both the transcriptional and post-transcriptional level (Dusane et al., 2010; Abdel-Mawgoud et al., 2011; Reis et al., 2011). Factors that were shown to play a key role in biosurfactants production include quorum sensing and stress conditions like nutrients deprivation. This might explain the influence of the sulfur source on the expression of biosurfactants-related genes. The cells produce biosurfactants to overcome the low bioavailability of some essential nutrients like sulfur. Obviously, DBT is less accessible than inorganic sulfate (water soluble) and DBT-sulfone (more polar than DBT).

Although the literature lacks comprehensive studies addressing the influence of the sulfur source on biosurfactants production, other essential nutrients were shown to be involved. For instance, biosurfactants production increases under nitrogen-limiting conditions (Abdel-Mawgoud et al., 2011; Reis et al., 2011). The nitrogen source indirectly affects the *rhlAB* operon via the nitrogen metabolism sigma factor regulator RpoN. Moreover, RpoN has a direct impact on the biosurfactants biosynthesis regulator RhlR. Also phosphate deprivation upregulates RhlR-controlled genes including those related to rhamnolipid biosurfactants production (Reis et al., 2011).

Based on the results of this study, it is evident that the sulfur source affects the transcriptional regulation of the *rhlABC* genes. Nonetheless, our data do not show if the regulation mechanism is direct or indirect. Moreover, it is not clear whether the sulfur source itself or a metabolite thereof is involved in the regulation process. Since the change in biosurfactants production occurred early in the exponential phase, it can be proposed that the sulfur substrate *per se* provoked this change.

Variations in biosurfactants productivity due to the provision of different sulfur sources can be perceived as a part of a global response of AK6U to sulfate starvation. This can be reconciled in terms of the difference in bioavailability of DBT, DBT-sulfone and MgSO_4_. In the presence of DBT as a sole sulfur source, the cells are stressed due to the very low aqueous solubility of DBT (sulfur-limiting conditions). Consequently, they produce biosurfactants to facilitate the uptake and sulfur utilization process.

Bacteria growing in the absence of easily accessible sulfur sources respond by synthesizing specific sulfate-starvation induced proteins (Kertesz and Wietek, 2001; Tralau et al., 2007). These include proteins related to the uptake and utilization of different sulfur sources, high-affinity transport systems for sulfate and cysteine, as well as antioxidants. Expression of the genes related to sulfate starvation is under the control of the global regulators (sigma factors) RpoN and RpoS (Tralau et al., 2007). Interestingly, both of these proteins play a role in the regulation of the biosurfactants production genes in *P. aeruginosa* (Reis et al., 2011). This may highlight the link between sulfur metabolism and biosurfactants production.

Considering the close structural similarity between DBT and DBT-sulfone, one might expect to see similar biosurfactants production profiles in cultures containing either of them as a sulfur source. Since this was not the case, we assume that AK6U has a well controlled sulfur source-mediated regulatory mechanism. This enables the cell to efficiently sense the bioavailability of the sulfur substrate and fine-tune biosurfactants production according to its needs.

The sulfur source can play a role in biosurfactants production via transcriptional regulation of the *rhlABC* genes. The AK6U strain appears to have the ability to fine-tune biosurfactants production according to the bioavailability of sulfur. AK6U overcomes the low aqueous solubility of DBT by producing biosurfactants. Overall, our findings provide a template for further studies aiming at better understanding of the underlying mechanisms through which the sulfur source influences biosurfactants production. This should have an impact on the industrial production of biosurfactants as well as other biotechnology-based processes such as biodesulfurization and bioremediation.

## Conflict of Interest Statement

The authors declare that the research was conducted in the absence of any commercial or financial relationships that could be construed as a potential conflict of interest.
